# Independent effects of white matter hyperintensities on cognitive, neuropsychiatric, and functional decline: a longitudinal investigation using the National Alzheimer’s Coordinating Center Uniform Data Set

**DOI:** 10.1186/s13195-019-0521-0

**Published:** 2019-07-27

**Authors:** Christian Puzo, Caroline Labriola, Michael A. Sugarman, Yorghos Tripodis, Brett Martin, Joseph N. Palmisano, Eric G. Steinberg, Thor D. Stein, Neil W. Kowall, Ann C. McKee, Jesse Mez, Ronald J. Killiany, Robert A. Stern, Michael L. Alosco

**Affiliations:** 1 Boston University Alzheimer’s Disease Center and CTE Center, Boston University School of Medicine, 72 E. Concord Street, Suite B7800, Boston, MA 02118 USA; 2 Department of Biostatistics, Boston University School of Public Health, Boston, MA USA; 3 Biostatistics and Epidemiology Data Analytics Center, Boston University School of Public Health, Boston, MA USA; 4 Department of Neurology, Boston University School of Medicine, Boston, MA USA; 5 Department of Pathology and Laboratory Medicine, Boston University School of Medicine, Boston, USA; 6 VA Boston Healthcare System, U.S. Department of Veteran Affairs, Jamaica Plain, USA; 7 Department of Anatomy and Neurobiology, Boston University School of Medicine, Boston, USA; 8 Center for Biomedical Imaging, Boston University School of Medicine, Boston, USA; 9 Departments of Neurosurgery and Anatomy & Neurobiology, Boston University School of Medicine, Boston, MA USA

**Keywords:** Alzheimer’s disease, Cerebral small vessel disease, Cerebrovascular disease, Executive function, Mild cognitive impairment, Preclinical, White matter hyperintensities

## Abstract

**Background:**

Longitudinal investigations are needed to improve understanding of the contributions of cerebral small vessel disease to the clinical manifestation of Alzheimer’s disease, particularly in the early disease stages. This study leveraged the National Alzheimer’s Coordinating Center Uniform Data Set to longitudinally examine the association between white matter hyperintensities and neuropsychological, neuropsychiatric, and functional decline among participants with normal cognition.

**Methods:**

The sample included 465 participants from the National Alzheimer’s Coordinating Center Uniform Data Set who had quantitated volume of white matter hyperintensities from fluid-attenuated inversion recovery MRI, had normal cognition at the time of their MRI, and were administered the National Alzheimer’s Coordinating Center Uniform Data Set neuropsychological test battery within 1 year of study evaluation and had at least two post-MRI time points of clinical data. Neuropsychiatric status was assessed by the Geriatric Depression Scale-15 and Neuropsychiatric Inventory-Questionnaire. Clinical Dementia Rating Sum of Boxes defined functional status. For participants subsequently diagnosed with mild cognitive impairment (MCI) or dementia, their impairment must have been attributed to Alzheimer’s disease (AD) to evaluate the relationships between WMH and the clinical presentation of AD.

**Results:**

Of the 465 participants, 56 converted to MCI or AD dementia (average follow-up = 5 years). Among the 465 participants, generalized estimating equations controlling for age, sex, race, education, *APOE ε4*, and total brain and hippocampal volume showed that higher baseline log-white matter hyperintensities predicted accelerated decline on the following neuropsychological tests in rank order of effect size: Trails B (*p* < 0.01), Digit Symbol Coding (*p* < 0.01), Logical Memory Immediate Recall (*p* = 0.02), Trail Making A (*p* < 0.01), and Semantic Fluency (*p* < 0.01). White matter hyperintensities predicted increases in Clinical Dementia Rating Sum of Boxes (*p* < 0.01) and Geriatric Depression Scale-15 scores (*p* = 0.01). Effect sizes were comparable to total brain and hippocampal volume. White matter hyperintensities did not predict diagnostic conversion. All effects also remained after including individuals with non-AD suspected etiologies for those who converted to MCI or dementia.

**Conclusions:**

In this baseline cognitively normal sample, greater white matter hyperintensities were associated with accelerated cognitive, neuropsychiatric, and functional decline independent of traditional risk factors and MRI biomarkers for Alzheimer’s disease.

## Introduction

Advances in the development of in vivo biomarkers of beta-amyloid (Aβ), tau, and neurodegeneration have increased the ability to accurately detect Alzheimer’s disease (AD) neuropathologic changes, even before onset of clinical symptoms [1–9]. In vivo biomarkers of specific (e.g., Aβ neuritic plaques) and non-specific (e.g., hippocampal atrophy) pathologies further assist with the study of disease mechanisms, monitoring of disease progression, and response to therapeutics. A popular target of clinical research into the pathogenesis of AD has been T2 fluid-attenuated inversion recovery (FLAIR) magnetic resonance imaging (MRI) white matter hyperintensities (WMH). WMH are non-specific biomarkers of WM pathology (e.g., gliosis, demyelination, axonal loss), but are often interpreted to reflect cerebral small vessel disease (CSVD) associated with aging and cardiovascular disease (CVD) [10–19]. CSVD is similarly a broad term that refers to pathologies that affect the cerebral arteries, arterioles, capillaries, and venules [20]. WMH are indeed often used to study the role of CSVD in the clinical and pathological manifestation of AD [10, 18, 21–31].

WMH may be evident on MRI long before symptom onset in patients with AD and may uniquely contribute to the clinical presentation of the disease. Lee et al. showed that participants who were autosomal-dominant for AD had increased burden of WMH 6 years prior to expected clinical onset of symptoms and that WMH were associated with Aβ levels in the cerebrospinal fluid (CSF) among carriers only [23]. Another study also reported WMH to demonstrate stronger associations with AD-specific pathology (e.g., Aβ) compared to traditional AD neuroimaging biomarkers (e.g., fluorodeoxyglucose [FDG]-PET, hippocampal volume) and cognitive test performance in cognitively normal controls from the AD Neuroimaging Initiative (ADNI) [21]. WMH commonly correlate with frontal lobe dysfunction [32–37] and CSVD is classically associated with impaired frontal-mediated cognitive systems [37–42]. CSVD and other white matter pathologies might contribute to the 10–40% of patients who have underlying AD pathology and present with an *initial* behavioral and/or dysexecutive syndrome [1, 43–45] and not a traditional amnestic profile.

Longitudinal studies are necessary to elucidate the role of WMH in the clinical manifestation of AD. There have been equivocal reports on the ability of WMH to predict diagnostic conversion. Several studies have shown that WMH predict diagnostic conversion across the AD clinical continuum [46–51]. In contrast, other studies have failed to observe WMH as a significant predictor of diagnostic conversion, particularly from mild cognitive impairment (MCI) to dementia [52–54]. Some of these inconsistencies may be related to differences in the measurement of WMH (e.g., quartiles versus continuous) [50], examination of baseline WMH compared to change in WMH over time [50], and/or the pathological heterogeneity of clinical AD [55]. Alternatively, if WMH are an early indicator of cognitive decline, they may be more sensitive to the subtle variations in neuropsychological test performance over time [41, 42, 56–58], specifically frontal systems functioning [35, 41, 42, 58], as opposed to diagnostic conversion.

Although WMH contribute to clinical heterogeneity in AD [35, 41, 42, 58], their effects in the early stages of disease (e.g., preclinical) are not well understood. Here, we leveraged the National Alzheimer’s Coordinating Center (NACC) Uniform Data Set (UDS) to conduct a longitudinal examination on the independent effects of FLAIR MRI WMH on neuropsychological, neuropsychiatric, and functional decline in participants who had normal cognition (NC) at baseline. These associations were examined after accounting for traditional risk factors and MRI biomarkers for AD, such as *APOE ε4* carrier status, total brain volume (TBV), and hippocampal volume. We hypothesized that greater baseline volume of WMH would be associated with an accelerated cognitive, neuropsychiatric, and functional decline.

## Methods

### Participants and design

**Fig. 1:**
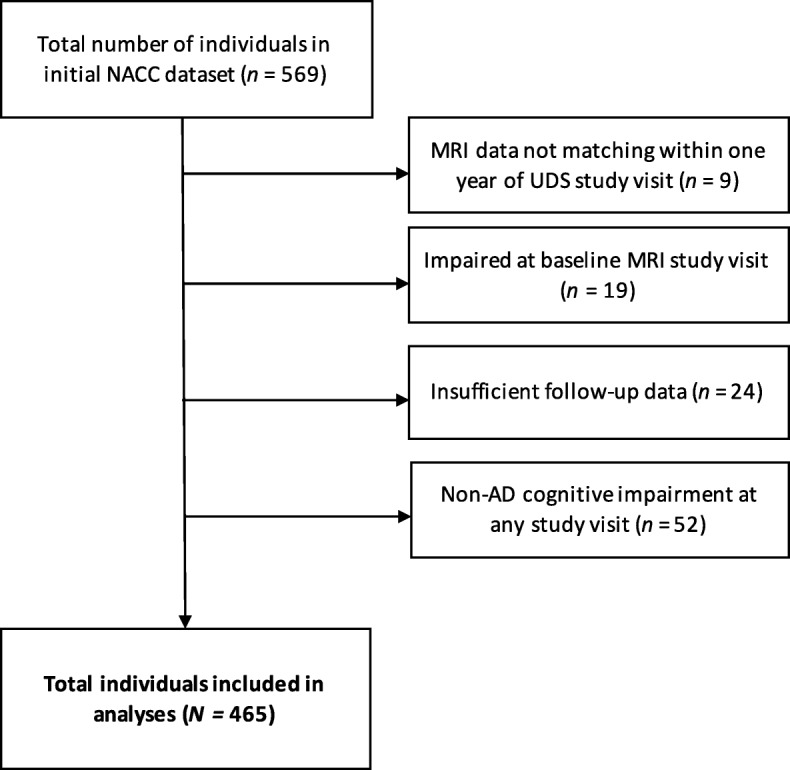
Participant flow chart. Abbreviations: AD = Alzheimer’s disease; CSF = cerebrospinal fluid; MRI = magnetic resonance imaging; NACC = National Alzheimer’s Coordinating Center

The sample included 465 participants from the NACC-UDS (see Fig. 1). The NACC was established in 1999 by the National Institute on Aging (NIA) to promote AD research by providing a publicly available database of clinical data gathered from NIA-funded Alzheimer’s Disease Centers (ADCs) across the USA. Since 2005, ADCs have contributed standardized cognitive, behavioral, and functional participant data from approximately annual study visits to a common database, known as the NACC-UDS [59–62]. A subset of ADCs also voluntarily submit structural MRI data [63] to NACC to include with the UDS. All ADCs that contribute data to NACC are approved by their local Institutional Review Board and participants provided informed consent at the ADC where they completed their study visits.

For this study, a formal data request was submitted to NACC to obtain UDS data for participants who were determined to have NC at their initial study visit, completed at least two follow-up visits, and had quantitated MRI data available, including total volume of T2 FLAIR WMH, at any time. The set includes data from the June 2018 NACC data freeze. The initial data set included 569 participants and the sample size was reduced to 465 after the following restrictions were applied: (1) the MRI must have been completed within 1 year of a NACC-UDS study visit and this was designated as the “baseline visit” for the purposes of this study (note that this did not necessarily correspond to the first overall NACC-UDS visit), (2) the participant must have been determined to have NC at the baseline MRI study visit, (3) participants must have had at least two follow-up visits following the baseline MRI visit, and (4) for participants subsequently diagnosed with MCI or dementia, their impairment must have been attributed to possible or probable AD to evaluate the relationships between WMH and the clinical presentation of AD. The final analytic sample of 465 participants included those who remained stable with NC from baseline, in addition to those who converted to MCI due to AD or AD dementia. There were 52 participants excluded for non-AD etiologies (*n* = 28 vascular disease, *n* = 3 Lewy body disease, *n* = 2 traumatic brain injury, *n* = 1 neoplasm, *n* = 1 normal pressure hydrocephalus, *n* = 3 depression, *n* = 3 other psychiatric disease, *n* = 4 medical illness, *n* = 1 medication effects, *n* = 6 other/unknown). Individuals with cognitive impairment due to non-AD causes were evaluated in a sensitivity analysis.

### Measures

#### Volumetric MRI

Volumetric MRI quantitation for NACC was conducted by the Imaging of Dementia & Aging (IDeA) Lab at the University of California Davis (Director: Charles DeCarli, M.D.; http://idealab.ucdavis.edu/). Total volume of WMH (in cm^3^) was calculated using T2 FLAIR scan files. Methods for WMH estimation were similar to those used for the ADNI-II [64]; a full description of methods can be found at https://www.alz.washington.edu/WEB/adni_proto.pdf and are described by Alosco et al. [63]. The TBV composite was calculated as the sum of total gray and white matter segmentations. Total hippocampal volume (a summary composite of the right and left hemispheres) was also calculated. TBV and hippocampal volume were included as covariates due to their sensitivity to clinical and pathological AD [50, 65].

#### Neuropsychological and neuropsychiatric functioning

All participants were administered a standardized battery of common neuropsychological tests at each study visit. These NACC-UDS tests are described elsewhere [59, 61, 62, 66]. Due to a recent change in the UDS neuropsychological test battery [62], the tests that have been most consistently administered over time were examined to maximize longitudinal data analyses. This resulted in 429 participants with UDS v.2.0 at baseline, with the remaining participants having had UDS v.1.0. Specific tests examined included the Mini-Mental State Examination (MMSE), Short Form of the Boston Naming Test (BNT), Animal Fluency and Vegetable Fluency (raw scores combined into one “Semantic Fluency” score), Trail Making Test (TMT) Parts A and B, Wechsler Adult Intelligence Scale-Revised Digit Symbol Coding (WAIS-R DSC), and Logical Memory Immediate (LM-IA) and Delayed Recall (LM-IIA). Lower scores reflect worse performance for all tests, with the exception of TMT A and B where lower scores are better.

Neuropsychiatric functioning was assessed at each study visit using the 15-item Geriatric Depression Scale (GDS-15) and the Neuropsychiatric Inventory-Questionnaire (NPI-Q). The GDS-15 is a self-report measure of 15 yes/no questions pertaining to the presence of depression symptoms. Scores range from 0 to 15 and higher scores represent greater depressive symptoms. The NPI-Q asks study partners to evaluate the presence or absence of 12 neuropsychiatric symptoms over the past month. Each symptom is rated on a scale from 0 (absent) to 3 (severe). Symptoms were separated into four domains based on the result of a factor analysis from a sample of outpatients with dementia [67]: hyperactivity (agitation, euphoria, irritability, disinhibition, aberrant motor behavior), psychotic symptoms (delusions, hallucinations), mood (depression, apathy, nighttime behavior disturbances, appetite/eating abnormalities), and anxiety (single anxiety item).

#### Cognitive diagnoses and dementia severity

Clinical research diagnoses of cognitive status (i.e., NC; cognitively impaired, not MCI; MCI; dementia) were made at each UDS visit using established criteria for MCI [68, 69] and AD dementia [70, 71]. For versions 1 and 2 of the UDS, MCI due to AD and AD dementia diagnoses were based on the National Institute of Neurological and Communicative Disorders and Stroke (NINCDS) and the Alzheimer’s Disease and Related Disorders Association (ADRDA) diagnostic criteria [71]. The Clinical Diagnosis Form D1 was updated in version 3 of the UDS [62] to adopt the 2011 National Institute on Aging-Alzheimer’s Association (NIA-AA) criteria for MCI due to AD [69] and AD dementia [70]. For the current sample, baseline clinical AD diagnoses were based on the UDS versions 1 and 2 NINCDS/ADRDA criteria (although all participants in this sample were cognitively normal at baseline). A subset of the follow-up clinical AD diagnoses were also made using the updated NIA-AA criteria. The NACC created a derived variable (i.e., NACCALZD) to harmonize UDS versions 1–3, and this variable was used to determine AD as the suspected etiology for those who converted at follow-up UDS visits. All diagnoses were based on available UDS data, including neuropsychological, neurological, and neuropsychiatric test results, and, when available, neuroimaging. Sum of Boxes from the CDR® Dementia Staging Instrument assessed the overall severity of clinical impairment [72, 73].

#### Demographic, medical, and genotype characteristics

Demographic and medical characteristics were ascertained during approximately annual UDS clinical evaluations. Blood samples are collected by the individual ADCs to determine *APOE ε4* allele status, which was dichotomized into *ε4* carriers versus non-carriers for the current analyses.

### Statistical analyses

Study hypotheses were tested using generalized linear models (GLM) estimated by generalized estimating equations (GEE) and binomial logistic regression models. Multiple linear regression models estimated by GEE were conducted using the “geepack” package [74] for R [75]. GEE using an autoregressive (AR1) correlation structure tested whether baseline volume of WMH predicted the slope of change over time of the following outcomes: (1) neuropsychological test scores: MMSE, Semantic Fluency, TMT-A and B, LM-IA and LM-IIA, BNT, and WAIS-R DSC, (2) CDR Sum of Boxes, and (3) GDS-15 and NPI-Q scores. All outcome raw scores were transformed into *z*-scores based on the performance of the sample at baseline in order to facilitate effect size comparisons between the variables of interest. All study visits were included in the GEE models. Binomial logistic regression models tested whether baseline total volume of WMH predicted conversion from NC to MCI due to AD at any time point; an insufficient number of participants progressed from NC to AD dementia to warrant reliable analyses. All models included all participants (*N* = 465).

For all models, the WMH variable was log-transformed due to a positively skewed distribution. Age at the time of the MRI scan, sex, race (White versus other), years of education, *APOE ε4* carrier status, TBV, and hippocampal volume were included as covariates in all GEE and logistic regression models. The baseline score on the relevant outcome variable of interest was included in all models. The main predictor variable of interest was the interaction between volume of WMH and the amount of time (in years) elapsed since the baseline MRI. Time since baseline in the models was included to facilitate examination of WMH and the slope of these variables over time, as well as account for differences in follow-up length and potential learning effects on the neuropsychological tests. Previous research has used total number of visits to test for learning effects in the UDS battery [66, 76]. Time since baseline is highly correlated with the number of UDS visits (*r* = 0.97); therefore, it was used to account for the total number of times an individual was exposed to the neuropsychological tests. Total number of visits was not also included due to violation of multicollinearity assumptions because of its correlation with time since baseline. The interpretation of the coefficients is based on SD unit increases between WMH and the corresponding outcome. *p* values for WMH effects from the GEE models were false discovery rate (FDR)-adjusted to the number of outcomes examined. For statistically significant findings, effects were illustrated graphically by separating participants into quartiles of WMH at baseline.

A series of sensitivity analyses were conducted. Because symptoms of depression are associated with WMH [77] and neuropsychological test performance [78], analyses were repeated with GDS-15 scores as a covariate to determine potential confounding from depression symptom severity. Analyses were also repeated with CVD risk factors associated with CSVD (i.e., systolic blood pressure, and diagnostic history of hypertension, diabetes, and hypercholesterolemia) as covariates to determine their potential confounding. Lastly, all analyses were repeated with intracranial volume entered as a covariate instead of TBV.

## Results

Participant demographics are shown in Table 1. All participants had a diagnosis of NC at the baseline MRI visit and were followed for an average of 5 years. A total of 56 participants (12.0%) had a diagnosis of MCI due to AD or AD dementia at any subsequent study visit; 51 participants converted from NC to MCI due to AD and 5 participants progressed directly from NC to AD dementia at consecutive study visits. Of the individuals who converted to MCI due to AD, 92.2% (47 of 51) had a diagnosis of amnestic MCI. There were eight participants who reverted from MCI to NC. Results for diagnostic conversion as an outcome remained unchanged without these participants included and therefore these participants were not excluded.

**Table 1:** Sample demographic and clinical characteristics. The total sample included 465 participants from the National Alzheimer’s Coordinating Uniform Data Set. All participants were diagnosed with normal cognition at the baseline study visit. The final analytic sample included those who remained stable with normal cognition and those who converted to MCI due to AD or AD dementia (*n* = 56). *APOE* data were missing for six participants

Variable	Mean (SD) or *n* (%)	Range
Age at baseline MRI visit	68.9 (11.8)	45–100
Total length of follow-up (years)	5.10 (2.14)	1.45–11.67
Total number of follow-up visits	4.62 (1.91)	2–10
Sex	146 (31.4%) male	
Years of education	15.37 (3.41)	1–25
Ethnicity	86.9% white, 10.3% AA, 2.8% other	
*APOE ε4* carrier status	162 (35.3%) carriers	
Systolic blood pressure	132 (18.8)	78–198
History of hypertension	200 (43.0%)	
History of diabetes	80 (17.2%)	
History of hypercholesterolemia	224 (48.2%)	
WMH volume at baseline (cm^3^)	4.70 (8.71)	0–61.24
Natural log of WMH at baseline	0.45 (1.52)	− 2.30–4.12
Diagnosis at final study visit		
Normal cognition	417 (89.7%)	
MCI	22 (4.7%)	
Dementia	26 (5.6%)	
CDR Sum of Boxes at final study visit	0.55 (1.83)	0–18

*Abbreviations*: *Aβ* beta-amyloid, *CDR* Clinical Dementia Rating scale, *MCI* mild cognitive impairment, *MRI* magnetic resonance imaging, *WMH* white matter hyperintensities

### Neuropsychological, neuropsychiatric, and functional decline

**Fig. 2:**
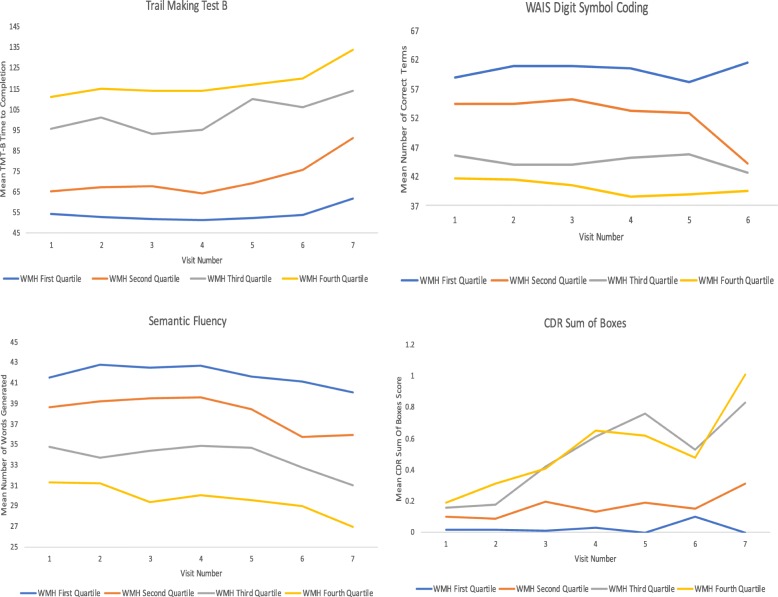
Mean neuropsychological test performance over time by white matter hyperintensity quartiles. The figure shows selected associations between white matter hyperintensity quartiles and clinical measures. Comparisons were made between quartiles of WMH (using raw values) amount at baseline. Higher quartiles reflect greater burden of white matter hyperintensities. Scores shown on the *y*-axis are raw neuropsychological test scores. For Trail Making Test B, higher scores reflect worse performance (i.e., WMH fourth quartile performed the worse). For all other measures, higher scores reflect better performance. For Trail Making Test B, individuals in the highest two quartiles (i.e., greatest WMH burden) experienced a mean increase of 2.9 s per year, whereas individuals in the lowest quartile only slowed by 0.9 s per year. On the WAIS Digit Symbol Coding, the highest quartile had a mean decline of 0.58, whereas the lowest quartile experienced a mean score increase of 0.11 per year. The mean slope for decline in Semantic Fluency was twice as steep among participants in the highest WMH quartile compared to the lowest WMH quartile (mean raw score decline of 0.61 words compared to 0.30 per year, respectively). For CDR Sum of Boxes, the participants in the lowest WMH quartile had a net slope of zero over time, whereas participants in the highest two quartiles had a mean raw score increase of 0.11 per year. GEE models showed the displayed relationships to be statistically significant after controlling for age, sex, race, years of education, *APOE ε4* carrier status, total brain volume, and hippocampal volume. Although not displayed, GEE models also showed statistically significant findings for Trail Making Test A, Logical Memory Immediate and Delayed Recall, and the 15-item Geriatric Depression Scale

Table 2 displays the results of GLM estimated by GEE. Only the results for the interaction between WMH and time since baseline are shown to demonstrate the role of WMH in predicting the slope of subsequent change. Higher baseline WMH were associated with decline on the following neuropsychological tests, in rank order of effect size magnitude: TMT-B (beta = 0.04) and WAIS-R DSC (beta = − 0.04), LM-IA (beta = − 0.04), TMT-A (beta = 0.03), and Semantic Fluency (beta = − 0.03); there was a non-significant trend for LM-IIA (beta = − 0.03, *p* = 0.05). There were no statistically significant effects for the MMSE or the BNT. For TMT-B and WAIS-R DSC, every one SD unit increase in baseline volume of WMH corresponded to a 0.04-unit SD decline in test performance per year. Figure 2 provides graphical representations of notable findings based on baseline WMH quartiles. For TMT-B, individuals in the highest two quartiles (i.e., greatest WMH burden) experienced a mean increase of 2.9 s per year, whereas individuals in the lowest quartile only slowed by 0.9 s per year. On the WAIS-R DSC, the highest quartile had a mean decline of 0.58, whereas the lowest quartile experienced a mean score increase of 0.11 per year. The mean slope for decline in Semantic Fluency was twice as steep among participants in the highest WMH quartile compared to the lowest WMH quartile (mean raw score decline of 0.61 words compared to 0.30 per year, respectively). Note that scores on the Logical Memory Immediate and Delayed Recall improved over time across all quartiles (likely due to the commonly observed practice effects [76]), but the rate of improvement was less for participants in the higher WMH quartiles.

**Table 2:** Summary of results from the generalized linear models estimated by generalized estimating equations (GEE). Only the results for the interaction effect for WMH and the time since baseline are displayed as a demonstration of the role of WMH in predicting the slope of these variables over time. The interpretation of the standardized beta coefficients is based on SD unit increases between log-transformed WMH and corresponding outcomes. All outcomes were raw scores that were transformed into *z*-scores based on the performance of the sample at baseline in order to facilitate effect size comparisons between the variables of interest. For example, for the MMSE, for every one SD unit increase in WMH, MMSE raw scores declined by 0.16 SD units per year. Age at baseline, race, sex, years of education, *APOE ε4* carrier status, baseline hippocampal volume, baseline total brain volume, and the baseline score on the variable of interest were included as covariates in all models. All *p* values are FDR-adjusted

Variable	*B*	SE	*p* value
Clinical status			
CDR Sum of Boxes	0.126	0.035	*< 0.01*
Neuropsychological tests			
MMSE	− 0.016	0.016	0.38
Semantic Fluency	− 0.031	0.007	*< 0.01*
TMT-A	0.031	0.007	*< 0.01*
TMT-B	0.040	0.007	*< 0.01*
LM-IA	− 0.040	0.015	*0.02*
LM-IIA	− 0.034	0.015	0.05
BNT	− 0.002	0.014	0.91
WAIS-R DSC	− 0.040	0.009	*< 0.01*
Neuropsychiatric symptoms			
GDS-15	0.040	0.014	*0.01*
NPI-Q Hyperactivity	0.022	0.015	0.23
NPI-Q Mood	0.014	0.011	0.27
NPI-Q Psychosis	0.025	0.016	0.19
NPI-Q Anxiety	0.007	0.014	0.66

*Abbreviations*: *BNT* Boston Naming Test Short Form, *CDR* Clinical Dementia Rating scale, *GDS-15* 15-item Geriatric Depression Scale, *LM-IA* Logical Memory Immediate Recall, *LM-IIA* Logical Memory Delayed Recall, *MMSE* Mini-Mental State Examination, *NPI-Q* Neuropsychiatric Inventory-Questionnaire, *TMT-A* Trail Making Test Part A, *TMT-B* Trail Making Test Part B, *WAIS-R DSC* Wechsler Adult Intelligence Scale-Revised Digit Symbol Coding

Higher baseline volume of WMH corresponded to increases on the GDS-15 over time (*p* = 0.01). There were no significant effects for the NPI-Q sub-scores (*p*s > 0.10). Greater volume of WMH at baseline predicted increased scores on the CDR Sum of Boxes over time (*p* < 0.01). The participants in the lowest WMH quartile had a net slope of zero over time, whereas participants in the highest two quartiles had a mean raw score increase of 0.11 per year. However, after accounting for demographics, *APOE ε4* carrier status, and length of follow-up for each participant, WMH were not a significant predictor of diagnostic change to MCI due to AD/AD dementia (*p* = 0.86). Similar null effects were observed when WMH quartiles were entered into the model rather than as a continuous variable.

The 52 individuals who were excluded from initial analyses due to non-AD impairment (for those who converted) were significantly older at baseline (mean age 75.5 years, *p* < 0.001). In sensitivity analyses that included individuals with impairment due to any etiology (i.e., AD as well as the 52 participants with other suspected pathologies), level of statistical significance remained across all neuropsychological, neuropsychiatric, and functional outcomes; in addition, the above trend for LM-IIA became statistically significant (*p* = 0.02). WMH were still not a significant predictor of conversion to amnestic MCI (*n =* 58) or non-amnestic MCI (*n* = 26).

### Post hoc: comparison of WMH, hippocampal volume, and total brain volume

Hippocampal volume and TBV were included as covariates in the above models to determine if WMH predicted the outcomes independently of these MRI-derived volumetric measures. We performed additional analyses to compare their predictive validity on cognitive change relative to WMH, i.e., we sought to compare effect sizes across the three variables to determine if the effects of WMH are of a similar magnitude. All three variables were transformed into standardized values (i.e., *z*-scores) based on the baseline scores, and separate models were performed predicting the change in each of the clinical outcomes that demonstrated statistically significant effects with WMH above (Table 3). Age at baseline, race, sex, years of education, *APOE ε4* carrier status, and the baseline value of the clinical outcome were included as covariates in models. The effect sizes for WMH were of comparable magnitude compared to TBV and hippocampal volume.

**Table 3:** Post hoc comparisons of the standardized coefficients for WMH, hippocampal volume, and total brain volume in neuropsychological, neuropsychiatric, and functional decline. Standardized beta coefficients are displayed for the interaction effect between the variable and time since baseline. All outcome raw scores were transformed into *z*-scores based on the performance of the sample at baseline in order to facilitate effect size comparisons between the variables of interest. The interpretation of the standardized beta coefficients is based on SD unit increases between MRI-derived volumetric measure (i.e., log-transformed WMH, hippocampal volume, total brain volume) and the corresponding outcomes age at baseline, sex, race, years of education, and *APOE ε4* carrier status were included as covariates in each model. The overlap in the absolute values of the confidence intervals demonstrated that no significant differences were observed between the three variables

Variable	Beta	95% CI	*p* value
CDR Sum of Boxes			
Log-transformed WMH	0.12	[0.06, 0.19]	*< 0.01*
Hippocampal volume	− 0.19	[− 0.29, − 0.07]	*< 0.01*
Total brain volume	− 0.15	[− 0.23, − 0.06]	*< 0.01*
Semantic Fluency			
Log-transformed WMH	− 0.03	[− 0.04, − 0.02]	*< 0.01*
Hippocampal volume	0.02	[0.01, 0.04]	*< 0.01*
Total brain volume	0.02	[0.01, 0.04]	*< 0.01*
TMT-A			
Log-transformed WMH	0.03	[0.02, 0.04]	*< 0.01*
Hippocampal volume	− 0.03	[− 0.04, − 0.01]	*< 0.01*
Total brain volume	− 0.02	[− 0.04, − 0.01]	*< 0.01*
TMT-B			
Log-transformed WMH	0.04	[0.02, 0.05]	*< 0.01*
Hippocampal volume	− 0.03	[− 0.05, − 0.01]	*< 0.01*
Total brain volume	− 0.03	[− 0.05, − 0.01]	*< 0.01*
LM-IA			
Log-transformed WMH	− 0.04	[− 0.07, − 0.01]	*0.01*
Hippocampal volume	0.03	[0.01, 0.06]	*0.01*
Total brain volume	0.02	[− 0.02, 0.04]	0.23
LM-IIA			
Log-transformed WMH	− 0.03	[0.06, − 0.00]	*0.02*
Hippocampal volume	0.04	[0.01, 0.06]	*0.01*
Total brain volume	0.01	[− 0.02, 0.03]	0.50
WAIS-R DSC			
Log-transformed WMH	− 0.04	[− 0.06, − 0.02]	*< 0.01*
Hippocampal volume	0.02	[0.00, 0.03]	*0.03*
Total brain volume	0.02	[0.01, 0.04]	*0.01*
GDS-15			
Log-transformed WMH	0.02	[0.01, 0.04]	*< 0.01*
Hippocampal volume	− 0.01	[− 0.03, 0.00]	0.08
Total brain volume	− 0.01	[− 0.03, 0.02]	0.61

### Post hoc: depression, cardiovascular disease risk factors, and intracranial volume

Effects for volume of WMH on neuropsychological test performance and diagnostic conversion remained when GDS-15 scores were included as covariates (data not shown). Statistical significance remained unchanged when systolic blood pressure and diagnostic history of hypertension, diabetes, and hypercholesterolemia were entered as additional covariates (data not shown). Statistical significance (including analyses for hippocampal volume) again remained unchanged when ICV was included as a covariate (data not shown).

## Discussion

Among participants from the NACC-UDS who were cognitively normal at baseline, higher baseline volume of WMH predicted an accelerated decline in executive function, attention and psychomotor speed, verbal learning and memory, and semantic fluency. Greater volume of baseline WMH additionally corresponded to accelerated functional decline and increases in self-reported symptoms of depression over time. All effects were independent of baseline age, sex, race, years of education, *APOE ε4* carrier status, TBV, and hippocampal volume, as well as CVD risk factors. The magnitude of effects for WMH was comparable to TBV and hippocampal volume. Baseline volume of WMH did not predict increased odds for diagnostic conversion. Overall, these findings suggest that WMH are an early and independent correlate of cognitive, neuropsychiatric, and functional decline.

For neuropsychological tests, baseline volume of WMH had the strongest effects for accelerated decline on measures that assess cognitive flexibility (TMT-B), working memory and information processing speed (WAIS-R DSC), and learning and encoding (LM Immediate Recall). LM scores actually improved over time, reflecting the sensitivity of this test to learning effects [76], although rate of improvement was slowest for those in the highest WMH quartile (learning effects are discussed further below). It is also noteworthy that WMH demonstrated an association with Semantic Fluency, but not BNT. This could reflect the greater variability of scores for Semantic Fluency. Alternatively, while both assess semantic memory, semantic fluency tasks have a significant executive function loading [79] that may have driven the effects for WMH. In addition to the effects of WMH on neuropsychological testing, baseline volume of WMH also predicted increases on the CDR Sum of Boxes over time, suggesting possible contribution of WMH to declines in everyday functioning. Similar findings were reported in 1290 individuals from the Northern Manhattan Study (NOMAS) [80]. The association between WMH and worsening self-reported symptoms of depression is similar to previous research from the Framingham Heart Study [77] and consistent with the known association between vascular disease and depression [81, 82].

The effects for WMH on the neuropsychological, neuropsychiatric, and functional measures were independent of, and comparable in magnitude to, TBV and hippocampal volume. Previous findings from Brickman et al. showed that volume of WMH had greater predictive validity for incident dementia relative to hippocampal volume [47]. Among older adults with NC from ADNI, volume of WMH and *APOE ε4* status were more strongly associated with cortical amyloid status on PET compared to other traditional biomarkers of AD, including FDG-PET and hippocampal volume [21]. WMH on MRI seem to be capturing unique pathophysiological processes and represents a sensitive (and practical) method for the early detection of cognitive and neuropsychiatric decline, monitoring of disease progression, and the study of white matter pathways in aging and neurodegeneration.

Baseline volume of WMH did not independently predict diagnostic conversion to MCI. In contrast, WMH predicted change for each neuropsychological test score investigated independent of age. The clinical meaningfulness is unclear due to the relatively small effect sizes as can be observed in Fig. 2 that shows the relationship between WMH quartiles and raw neuropsychological test performance. As an example, for Trail Making Test B, individuals in the highest two quartiles (i.e., greatest WMH burden) experienced a mean increase of 2.9 s per year, whereas individuals in the lowest quartile slowed by 0.9 s per year. The relatively small effect sizes are expected given the sample included participants with baseline NC where there are only subtle, if any, cognitive, neuropsychiatric, and functional impairments and changes. Examination of continuous neuropsychological test scores is perhaps optimal for the detection of brain behavior relationships particularly in the absence or early stages of disease.

The current study used FLAIR WMH to provide further insight into the complicated role of CSVD in early cognitive decline. Greater than 50% of dementia cases throughout the world are associated with CSVD [18, 83–86] and 80% of patients diagnosed with AD have cerebrovascular disease pathology at autopsy [31]. CSVD is a broad term that refers to pathologies of the cerebral arteries, arterioles, capillaries, and venules [20]. WMH are widely supported to be markers of CSVD associated with aging and CVD [10–17]. Yet, our sensitivity analyses showed that the effects of WMH remained after controlling for CVD risk factors (i.e., systolic blood pressure, diagnostic history of hypertension, diabetes, and hypercholesterolemia). Although assessments of CVD and related lifestyle behaviors (e.g., exercise, diet) were limited in this study, increasing research suggests that WMH represent white matter pathologies not solely due to CVD and could reflect white matter damage (e.g., axonal loss, demyelination) related to AD, even in the early stages of the disease [23, 87]. The underlying pathologies of WMH may also change throughout the progression of AD [20].

Regardless of etiology, WMH are known to affect the clinical expression of AD [88] and contribute to cognitive heterogeneity in the early stages of disease, including executive dysfunction [23, 89–91]. We propose that CSVD and/or other pathologies of the white matter may contribute to dysexecutive clinical presentations. Previous research has failed to detect an association between WMH and cognitive decline using memory-based measures [92]. The limitations of the NACC-UDS battery preclude the ability to disentangle a distinct executive cognitive profile. Only Logical Memory assessed episodic memory, and word list learning tasks are not part of the NACC-UDS. Other research has also shown that WMH correlates with amnestic MCI and continuous memory tests [93], although the extent to which these effects are frontal versus hippocampal-mediated is uncertain. Nonetheless, approximately 10–40% of patients with underlying AD neuropathologic changes present with an *initial* behavioral and dysexecutive syndrome as opposed to episodic memory impairment [1, 43–45]. This dysexecutive clinical phenotype of AD has been described elsewhere [1, 45, 94–96] and is associated with genetic risk factors [95] and the presence of co-morbid neurodegenerative disease pathologies (e.g., frontotemporal lobar degeneration [FTLD]) [45]. The dysexecutive phenotypes of clinical AD are associated with a more rapid progression [96, 97] that could be attenuated if its cause is due to modifiable risk factors.

There are several limitations to the present findings. The present study did not include biomarker assessment of underlying AD pathology, including amyloid or p-tau pathology. Only a few NACC ADCs contribute CSF biomarker data and there is no standardized protocol for the collection and biomarker analysis of CSF. The relationships between WMH and CSF [22] and PET [21, 98] biomarkers for AD have been reported previously. For this study, most participants remained cognitively normal, only 52 participants were excluded due to non-AD suspected etiologies (for those who converted), and statistically significant effects remained when these 52 participants were included. WMH might therefore have similar cognitive and neuropsychiatric effects, regardless of the underlying etiology.

Learning effects may have led to an underestimation of the effects of WMH on neuropsychological test performance over time. Yet, learning effects may also have had minimal influence on the estimated effects for several reasons. First, time since baseline (*r* = 0.97 with number of UDS visits) was included in the statistical models to account for differences in number of exposures to neuropsychological tests. Second, the longitudinal statistical models for neuropsychological test performance included all participants and all study visits. Therefore, if learning effects were present, those with low WMH burden would be expected to demonstrate improved performance over time, whereas participants with high WMH burden would show a smaller than expected learning curve. Such pattern would provide evidence for the detrimental effects of WMH and this was only observed for LM, i.e., scores on LM improved over time, but the rate of improvement was slowest for those with the most WMH burden. The LM test is known for its sensitivity to learning effects [76, 99–101]. Research in the NACC-UDS has shown that these learning effects become statistically and clinically significant at visit 6 and so on, and the current study had, on average, 5 years of follow-up visits. Aside from LM, other UDS tests have not been shown to have learning effects [66].

There is limited serial quantitated MRI data available from NACC, and it will be essential to leverage the NACC data set in the future to examine changes in WMH and its correspondence to neuropsychological, neuropsychiatric, and functional decline. We examined total WMH volume and evolving research shows there are differential regional effects for WMH in predicting cognitive outcomes [32, 47]. WMH were associated with GDS-15 scores, but not the NPI-Q. This is possibly due to differences in the symptom dimensions assessed and method of data reporting (patient versus informant-reported, respectively) between the two measures. The NPI-Q was also developed as an index of symptoms observed in dementia and may have lacked sensitivity in this sample of cognitively normal participants. Finally, NACC data is derived from convenience samples and participants are not randomly sampled from the general population. The participants and selection of participants are further different across the ADCs that contribute data to NACC, including some centers that might actually exclude participants based on vascular disease (e.g., stroke).

## Conclusions

Greater baseline volume of FLAIR MRI WMH was associated with neuropsychological, neuropsychiatric, and functional decline independent of traditional risk factors and MRI biomarkers for AD in this sample of participants with baseline NC from the NACC-UDS. The recent NIA-Alzheimer’s Association research framework emphasizes biomarkers of amyloid, tau, and neurodegeneration for the detection, diagnosis, and study of AD [3]. Integration of CSVD into diagnostic, pathogenic, and treatment models of AD might be indicated [3, 4, 88, 102], but disentangling the precise role of CSVD in the clinical and neuropathological expression of AD remains warranted.

## Data Availability

The data sets generated and/or analyzed during the current study are available through the publicly available National Alzheimer’s Coordinating Center UDS database. The current set includes data from the June 2018 NACC data freeze.

## Abbreviations

AD: Alzheimer’s disease
ADC: Alzheimer’s Disease Center
ADNI: Alzheimer’s Disease Neuroimaging Initiative
BNT: Boston Naming Test
CDR: Clinical Dementia Rating
CSF: Cerebrospinal fluid
CSVD: Cerebral small vessel disease
CVD: Cardiovascular disease
FDR: False discovery rate
FLAIR: Fluid-attenuated inversion recovery
GDS: Geriatric Depression Scale
GEE: Generalized estimating equations
LM: Logical Memory
MCI: Mild cognitive impairment
MMSE: Mini-Mental State Examination
MRI: Magnetic resonance imaging
NACC: National Alzheimer’s Coordinating Center
NC: Normal cognition
NPI-Q: Neuropsychiatric Inventory-Questionnaire
TBV: Total brain volume
TMT: Trail Making Test
UDS: Uniform Data Set
WAIS-R DSC: Wechsler Adult Intelligence Scale-Revised Digit Symbol Coding
WMH: White matter hyperintensities
